# WO_3_ Nanoplates Decorated with Au and SnO_2_ Nanoparticles for Real-Time Detection of Foodborne Pathogens

**DOI:** 10.3390/nano14080719

**Published:** 2024-04-19

**Authors:** Xueyan Li, Zeyi Wu, Xiangyu Song, Denghua Li, Jiajia Liu, Jiatao Zhang

**Affiliations:** 1 School of Materials Science and Engineering, Beijing Key Laboratory of Construction-Tailorable Advanced Functional Materials and Green Applications, Beijing Institute of Technology, Beijing 100081, Chinazhangjt@bit.edu.cn (J.Z.); 2 Key Laboratory of Agricultural Information Service Technology of Ministry of Agriculture, Agricultural Information Institute of Chinese Academy of Agricultural Sciences, Beijing 100081, China; 3 School of Chemistry and Chemical Engineering, MIIT Key Laboratory of Medical Molecule Science and Pharmaceutical Engineering, Beijing Institute of Technology, Beijing 100081, China; 4 MOE Key Laboratory of Cluster Science, Beijing Institute of Technology, Beijing 100081, China

**Keywords:** Au/SnO_2_–WO_3_ nanoplates, photochemical deposition, 3-hydroxy-2-butanone

## Abstract

Nowadays, metal oxide semiconductor gas sensors have diverse applications ranging from human health to smart agriculture with the development of Internet of Things (IoT) technologies. However, high operating temperatures and an unsatisfactory detection capability (high sensitivity, fast response/recovery speed, etc.) hinder their integration into the IoT. Herein, a ternary heterostructure was prepared by decorating WO_3_ nanoplates with Au and SnO_2_ nanoparticles through a facial photochemical deposition method. This was employed as a sensing material for 3-hydroxy-2-butanone (3H-2B), a biomarker of *Listeria monocytogenes*. These Au/SnO_2_–WO_3_ nanoplate-based sensors exhibited an excellent response (R_a_/R_g_ = 662) to 25 ppm 3H-2B, which was 24 times higher than that of pure WO_3_ nanoplates at 140 °C. Moreover, the 3H-2B sensor showed an ultrafast response and recovery speed to 25 ppm 3H-2B as well as high selectivity. These excellent sensing performances could be attributed to the rich Au/SnO_2_–WO_3_ active interfaces and the excellent transport of carriers in nanoplates. Furthermore, a wireless portable gas sensor equipped with the Au/SnO_2_–WO_3_ nanoplates was assembled, which was tested using 3H-2B with known concentrations to study the possibilities of real-time gas monitoring in food quality and safety.

## 1. Introduction

With the development of Internet of Things (IoT) technologies, gas sensors are in great demand for numerous applications, including food, medical (diagnosis), industry, environment, and agriculture [1,2]. To be integrated into the IoT, there is a strong need for advanced miniature gas sensors with a low cost, low power consumption, and excellent detection capabilities (high sensitivity, fast and sensitive response, etc.) to satisfy the demands of real-time monitoring [3]. Among the various gas sensors, metal oxide semiconductor (MOS)-based chemical-resistive gas sensors are being studied most actively due to their good stability, low cost, compact size, and high sensitivity, which make them a promising candidate for integration into the IoT [4]. However, their high operating temperature (above 300 °C) limits their utilization in the IoT.

Among the various MOSs, attention to tungsten oxide (WO_3_) has increased because its sensing performance mainly depends on the synthesis route, through which the sensing factors (active sites, microstructure, localized gas–solid interface, and so on) can be controlled [5,6,7,8]. Moreover, its crystalline lattice allows it to accommodate certain oxygen deficiencies, which could enhance its sensing performance by modulating the electronic band structure and increasing the carrier densities [9]. However, a single-component WO_3_-based sensing layer could hardly satisfy all requirements for sensitivity, selectivity, stability, and working temperatures. Diverse strategies, mainly consisting of morphology modulation [4], the construction of heterostructures [10,11], and the decoration of catalysts [6,12], have been developed to improve the gas-sensing capability of WO_3_ nanomaterials. Constructing composite materials has been put forward to create more gas adsorption sites and reduce the charge transport channel, thus enhancing the overall sensing performance. For example, Kaur et al. reported a selective sensing behavior toward both oxidizing and reducing gas analytes by employing NiO/NiWO_4_/WO_3_ heterojunctions [10]. In addition, decorating MOSs with noble metals (Ag, Au, Pd, and Pt) has been proposed as an effective method to promote the sensing performance at lower operating temperatures [13,14,15,16]. For instance, WO_3_ nanoflowers decorated with PtAu bimetallic nanocrystals led to a high response to 20 ppm n-pentanol at 200 °C, a response that was 105 times higher than that for pure WO_3_ [14]. As reported for Pt-anchored WO_3_ chemiresistors, the formation of surface-active sites and inward diffusion induced by a pulse temperature modulation strategy explained the superior response to trimethylamine and xylene [12]. More recently, Zhang et al. prepared Pd/Si–mWO_3_ microspheres, which exhibited good selectivity and sensitivity to hydrogen at 210 °C [6]. In addition to these classical heterojunctions built from two components, the construction of heterostructures by assembling different active components offers additional possibilities [17,18]. For example, Tammanoon et al. reported that PdOx–CuOx co-loaded porous WO_3_ microspheres were highly selective and sensitive to methyl mercaptan at 200 °C and, more importantly, the co-loading of PdOx with CuOx significantly improved the methyl mercaptan selectivity against hydrogen sulfide [19]. Overall, the unique heterointerface effect optimizes adsorption/reaction processes and the following charge transfer between the target gas and the sensing layer, thereby decreasing the working temperature and improving the sensing sensitivity. Despite the significant results that have been attained, it is still highly desirable to synthesize WO_3_ hybrid nanocomposites with fine-tuned surface/interface properties to meet future IoT environment monitoring demand.

*Listeria monocytogenes* is a highly contagious foodborne pathogen that causes listeriosis outbreaks primarily in immunocompromised individuals [13]. It can be found in various food sources such as vegetables, meat, seafood, and dairy products and has the ability to survive and even grow under refrigeration and other food preservation measures [20]. Infection from *Listeria monocytogenes* can lead to symptoms such as fever, nausea, diarrhea, and, in severe cases, bacteremia, complications, and meningitis [20]. According to the World Health Organization (WHO), although listeriosis is a relatively rare disease (with 0.1 to 10 cases per 1 million people per year, depending on geographical location), its high fatality rate makes it a significant public health concern [21]. For instance, a listeriosis outbreak in South Africa in 2018 resulted in 1024 infections and over 200 deaths [22], while another outbreak associated with enoki mushrooms affected 48 individuals in 2020 [23]. Conventional methods such as plate colony counts, biochemical tests, molecular assays, and immunoassays require professional operators and specialized equipment, and it requires a few days to a week to obtain results [24]. Therefore, there is an urgent need for the development of rapid, effective, and user-friendly techniques to detect *Listeria monocytogenes*. Recently, the detection of specific microbial volatile organic compounds (WVOCs) has emerged as a promising method for on-site pathogen detection and identification [25]. Among the WVOCs of *Listeria monocytogenes*, one is 3-hydroxy-2-butanone (3H-2B), which accounts for approximately a 32% abundance. Moreover, the concentration of 3H-2B exhibits a good linear relation with the incubation time of *Listeria monocytogenes* [26]. Since the initial exploration by Zhu et al., who investigated the feasibility of utilizing mesoporous WO_3_ nanomaterials for the selective detection of 3H-2B to monitor *Listeria monocytogenes*, various MOS nanomaterials with diverse morphologies have been employed to detect 3H-2B [26]. For instance, mesoporous WO_3_/Au nanocomposites prepared using a soft-template approach demonstrated a sensitive detection of 3H-2B (175 °C; R_a_/R_g_ = 18.8 to 2.5 ppm) [27]. Zeb et al. synthesized AuPdO-modified Cu-doped K_2_W_4_O_13_ nanowires via hydrothermal treatments, followed by in situ reduction and impregnation, which exhibited dual selectivity in detecting 3H-2B at 120 °C (R_a_/R_g_ = 242 to 10 ppm) and triethylamine at 200 °C, respectively [28]. Wang et al. developed a highly sensitive 3H-2B sensor based on zinc oxide nanorods decorated with Co_3_O_4_ nanoparticles, delivering an exceptional sensing performance (260 °C; R_a_/R_g_ = 550 to 5 ppm) [29]. However, these aforementioned nanomaterials still possess limitations in terms of their material synthetic complexity, response/recovery time, sensitivity, limit of detection, and selectivity, thus necessitating further efforts toward the development of more sensitive sensing materials with a facile synthesis method for the real-time tracing of *Listeria monocytogenes*.

Herein, a ternary heterostructure was prepared by decorating WO_3_ nanoplates with Au and SnO_2_ nanoparticles through a facial photochemical deposition method. These nanocomposite materials were carefully characterized in terms of morphology and structure and their gas sensitives to 3H-2B were measured. The underlying sensing mechanisms were discussed. Furthermore, a portable gas sensor loaded with the Au/SnO_2_–WO_3_ nanoplates was assembled, which was tested using 3H-2B with known concentrations to study the possibilities of real-time gas monitoring in food quality and safety.

## 2. Materials and Methods

The WO_3_ nanoplates were prepared via the hydrothermal and calcination method. Typically, 300 mg sodium tungstate (Sinopharm, Shanghai, China) was added to 15 mL deionized water and stirred for 10 min. Then, 0.45 mL L-lactic acid (Aladdin, Shanghai, China) and 0.675 mL of a 6 mol/L hydrochloric acid (Tgreag, Beijing, China) solution were added in order, with stirring after each addition. The resulting solution was heated at 120 °C for 12 h and a yellow-green powder was collected after washing and drying. Finally, the WO_3_ nanoplates were produced after calcination under an air atmosphere at 500 °C for 1 h. The deposition of Au or SnO_2_ nanoparticles on the surface of the WO_3_ nanoplates was carried out using the photochemical deposition method. Typically, 50 mg WO_3_ was dispersed in 40 mL deionized water. The mixture was stirred for 10 min and then we added X (X = 0.5, 0.75, 1, and 1.25) mL HAuCl_4_ (5 mg/mL) (Sinopharm, Shanghai, China) and SnCl_2_ (5 mg/mL) (Aladdin, Shanghai, China). Under continuous stirring and Xenon lamp irradiation for 4 h, a purple suspension was obtained. The purple suspension was centrifuged, washed, and dried to obtain purple powder samples, designated as XAu/SnO_2_–WO_3_ nanoplates. For comparison, Au–WO_3_ and SnO_2_–WO_3_ nanoplates were also prepared under the same deposition conditions from HAuCl_4_ or SnCl_2_ and KIO_3_ on the surface of WO_3_ nanoplates, respectively. The prepared samples are summarized in Table S1. Characterizations for all samples are shown in the Supplementary Materials.

A gas-response instrument (WS-30B, Weisheng Ltd., Zhengzhou, China) was used to measure the gas-sensing characteristics using the static gas distribution method. The procedure for the sensing film preparation and gas-sensing measurements is described in detail in the Supplementary Materials. The resistance ratio between the gas sensor in air (R_a_) and the target gas (R_g_) was calculated as the gas response, where R_g_/R_a_ and R_a_/R_g_ were the oxidizing gas and reducing gases, respectively. The response/recovery time was defined as the time for the variation in the gas response to reach 90% of the equilibrium value after a test gas was injected or released.

## 3. Results and Discussion

### 3.1. Morphology of WO_3_, SnO_2_–WO_3_, and XAu/SnO_2_–WO_3_ Nanoplates

**Figure 1:**
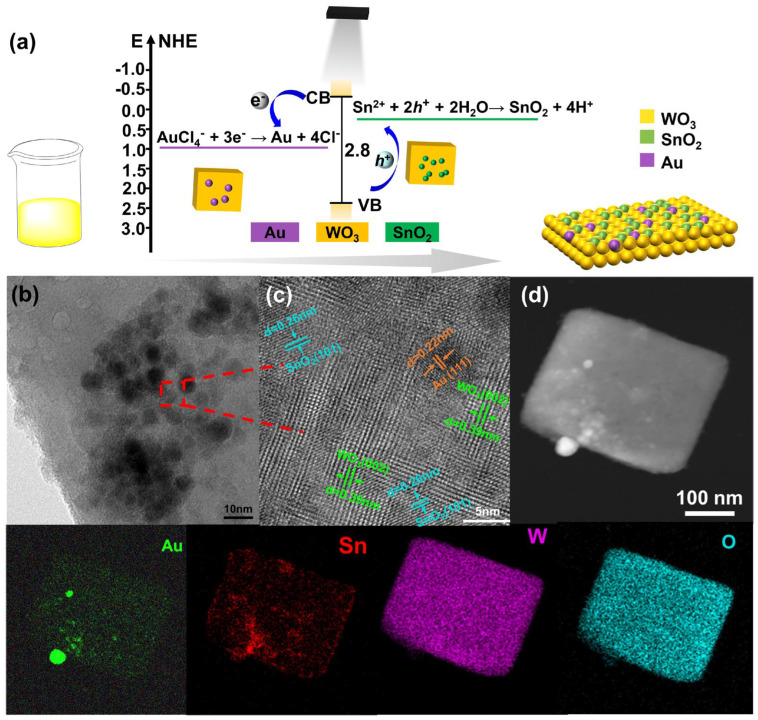
(**a**) The synthesis schematic of XAu/SnO_2_–WO_3_ nanoplates. (**b**,**c**) HRTEM images. (**d**) HAADF−STEM image and corresponding EDS elemental mapping results of the 1Au/SnO_2_–WO_3_ nanoplates.

The procedure for the synthesis of the XAu/SnO_2_–WO_3_ nanoplates is illustrated in Figure 1a. The WO_3_ nanoplates were initially synthesized using the hydrothermal and calcination method. As shown in Figure S1, WO_3_·H_2_O nanoplates were generated after a hydrothermal treatment. Samples calcinated at different temperature were characterized using TEM (Hitachi H-7650, Hitachi, Japan) and XRD (Bruker D8, Karlsruhe, German) techniques (Figure S2), and monoclinic WO_3_ nanoplates with a uniform size (200~300 nm) were selected for further modification. Finally, the Au and SnO_2_ nanoparticles were decorated on the WO_3_ nanoplates using the photochemical deposition method. Photochemical deposition is an effective method for co-catalyst deposition in the synthesis of photocatalysts, involving reductive and oxidative photodeposition by photogenerated electrons and photoinduced holes, respectively [30]. Generally, sacrificial electron donors/acceptors are required during reductive/oxidative photodeposition. Herein, Au and SnO_2_ nanoparticles were simultaneously photochemically deposited onto WO_3_ nanoplates using HAuCl_4_ and SnCl_2_ solutions as precursors. The morphology and structural observation of the obtained samples are shown in Figure 1b and Figure S3. The large dark spots on the WO_3_ nanoplates were believed to be Au nanoparticles, while the light chain-like nanoparticles were SnO_2_ nanoparticles (Figure S3). The HRTEM image in Figure 1c demonstrates that the Au and SnO_2_ nanoparticles were effectively decorated onto the WO_3_ nanoplates. The lattice spacings of 0.39 nm, 0.22 nm, and 0.26 nm matched well with the (002) crystal plane of WO_3_, the (111) plane of Au, and the (101) plane of SnO_2_, respectively, where the formation of heterogeneous interfaces resulted in a slight lattice distortion. The high-angle annular dark-field scanning TEM (HAADF-STEM) image and corresponding energy-dispersive X-ray spectroscopy (EDS) elemental mapping (Figure 1d) confirmed the homogeneous distribution of Au and Sn elements in the XAu/SnO_2_–WO_3_ nanoplates. Some large Au nanoparticles were found, similar to the samples obtained using the photochemical deposition method [31]. These results demonstrated the formation of XAu/SnO_2_–WO_3_ composites with an intensely coupled interaction between the individual components.

### 3.2. Microstructure of WO_3_, SnO_2_–WO_3_, and XAu/SnO_2_–WO_3_ Nanoplates

**Figure 2:**
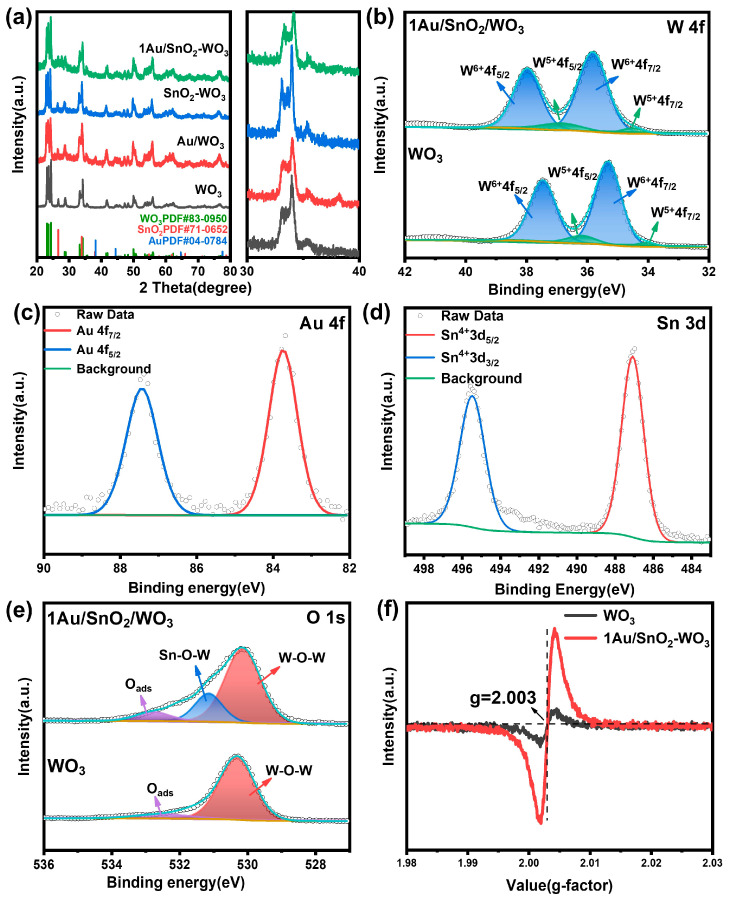
(**a**) XRD patterns of WO_3_ nanoplates, Au–WO_3_ nanoplates, SnO_2_–WO_3_ nanoplates, and 1Au/SnO_2_–WO_3_ nanoplates; XPS spectra of (**b**) W 4f, (**c**) Au 4f, (**d**) Sn 3d, and (**e**) O 1s; (**f**) EPR spectra of WO_3_ and 1Au/SnO_2_–WO_3_ nanoplates.

An XRD analysis was carried out to characterize the crystal structures of the as-prepared samples. As shown in Figure 2a and Figure S4a, the diffraction peaks of all the samples matched well with the monoclinic crystal structure of WO_3_ (JCPDS No. 83-0950). The characteristic peak of SnO_2_ at 33.887° only appeared in SnO_2_–WO_3_, corresponding with the (101) crystal plane of SnO_2_ (JCPDS No. 72-1147). For Au–WO_3_, the characteristic peak appeared at 38.184°, corresponding with the (111) crystal plane of Au (JCPDS No. 04-0784). However, there was no characteristic peaks of Au and SnO_2_ detected in the XAu/SnO_2_–WO_3_ nanoplates, which could be ascribed to the small loading amount of Au and SnO_2_.

X-ray photoelectron spectroscopy (XPS) measurements were used to further analyze the chemical state of the elements (Figure 2b–e and Figure S4b,c). The survey spectrum (Figure S4b) demonstrated the presence of W, Sn, Au, and O, indicating the high purity of the samples. Figure 2b compares the spectra of W 4f in WO_3_ and the 1Au/SnO_2_–WO_3_ nanoplates. The peaks of W^6+^ (35.81 eV and 37.7 eV) and W^5+^ (34.5 eV and 36.5 eV) shifted toward the high binding energy side. This indicated that there was a strong interaction between WO_3_, Au, and SnO_2_ and the electrons around the W atom decreased [32]. In the 4f spectrum of the Au element, the peaks at the binding energies of 83.86 eV and 87.54 eV matched well with Au 4f_7/2_ and Au 4f_5/2_, indicating that the Au element existed in the form of Au [33,34]. In the 3d spectrum of the Sn element, the peaks at the binding energies of 487.18 eV and 495.56 eV corresponded with Sn 3d_5/2_ and Sn 3d_5/2_, respectively, indicating that Sn existed in a +4 oxidation state [35]. The mass percentage of the Au and Sn elements is listed in Table S2. With an increase in the dosage, the content of the Au and Sn elements in the sample also increased. The O 1s XPS spectra of the WO_3_ and 1Au/SnO_2_–WO_3_ nanoplates (Figure 2e) could be deconvoluted into different peaks, corresponding with W–O–W (530.1 eV) [36], Sn–O–W (531.94 eV) [37], and adsorbed oxygen (O_ads_; 532.58 eV), respectively. The proportion of adsorbed O increased in the 1Au/SnO_2_–WO_3_ nanoplates, which could be ascribed to surface oxygen vacancies. The appearance of the Sn-O-W bond indicated that there was a good interfacial interaction between the SnO_2_ and WO_3_ phases.

An electron paramagnetic resonance (EPR) (Bruker A300-10/12, German)analysis was carried out to study the surface oxygen vacancies of the samples. As shown in Figure 2f, there was a relatively higher intensity peak at g ≈ 2.003 for the 1Au/SnO_2_–WO_3_ nanoplates than that of the WO_3_ nanoplates under the same conditions, indicating electron trapping at oxygen vacancies [38]. These results confirmed the presence of surface oxygen vacancies on the 1Au/SnO_2_–WO_3_ nanoplates, which correlated with the above XPS results. Figure S4d shows the Raman spectra of the WO_3_ and 1Au/SnO_2_–WO_3_ nanoplates. As the W-O bond changed due to the increase in oxygen vacancy, the peak of the 1Au/SnO_2_–WO_3_ nanoplates moved to a longer wavelength at 600 cm^−1^ and the peak intensity became weaker than that of the WO_3_ nanoplates.

### 3.3. Gas-Sensing Performance

**Figure 3:**
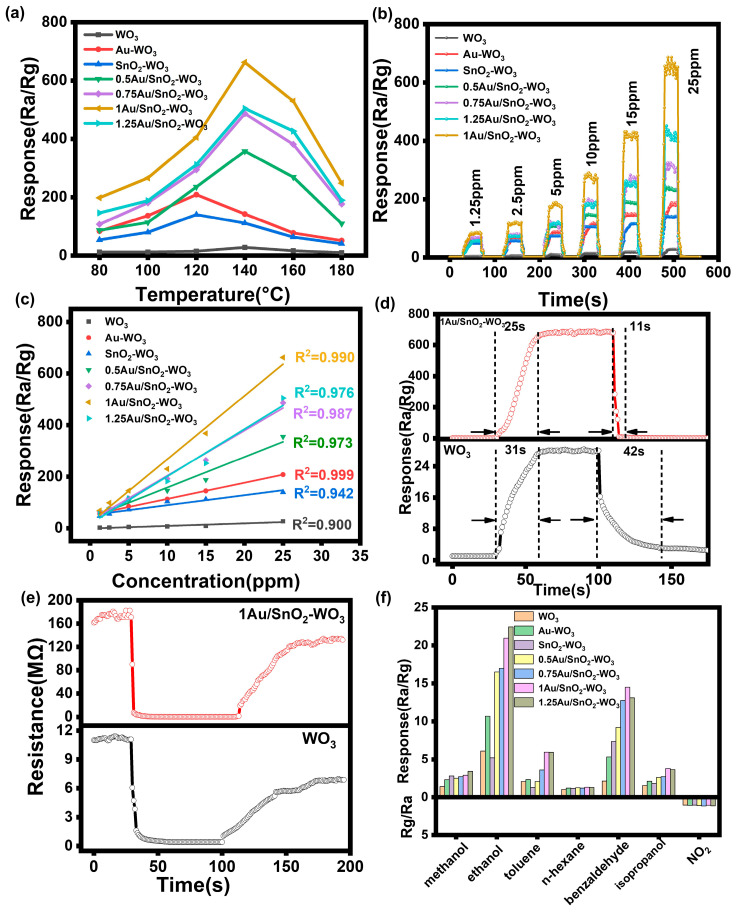
(**a**) Response curves of WO_3_ and XAu/SnO_2_–WO_3_ nanoplate-based sensors to 25 ppm 3H-2B at different working temperatures. (**b**) Dynamic response and recovery curves of WO_3_ and XAu/SnO_2_–WO_3_ nanoplate-based sensors toward different concentrations (1.25, 2.5, 5, 10, 15, and 25 ppm) of 3H-2B at 140 °C. (**c**) Response curves of WO_3_ and XAu/SnO_2_–WO_3_ nanoplate-based sensors toward different concentrations of 3H-2B. (**d**,**e**) Dynamic response and recovery curves of WO_3_ and XAu/SnO_2_–WO_3_ nanoplate-based sensors toward 25 ppm 3H-2B. (**f**) Selectivity tests of WO_3_ and XAu/SnO_2_–WO_3_ nanoplate-based sensors toward different target gases.

The gas-sensing performance of the as-prepared samples was carefully studied and compared. Given that the operating temperature strongly affects sensing performances, the optimum working temperatures of the sensors were first identified. As shown in Figure 3a, sensors based on the WO_3_, Au–WO_3_, SnO_2_–WO_3_, and XAu/SnO_2_–WO_3_ nanoplates exhibited a notable response in a 25 ppm 3-hydroxy-2-butanone (3H-2B, 98%; Aladdin, Shanghai, China) atmosphere within an operating temperature range of 80–180 °C. The response patterns of all the sensors displayed a similar trend of increasing before decreasing. Notably, the sensing response values of the XAu/SnO_2_–WO_3_ nanoplates to 25 ppm 3H-2B at 140 °C were significantly higher than those of the WO_3_, Au–WO_3_, and SnO_2_–WO_3_ sensors. Among them, the 1Au/SnO_2_–WO_3_ nanoplates showed the maximum response of 662 to 25 ppm 3H-2B at 140 °C, which was 24 times larger than that of the WO_3_ sensor. The working temperature was selected as 140 °C for the subsequent tests. With an increase in the deposition amount of Au and SnO_2_, the sensing response to the target gas increased, but the response value of 1.25Au/SnO_2_–WO_3_ to 3H-2B began to decrease due to the aggregation and growth of Au and SnO_2_ nanoparticles and the reduction in active sites. The detectivity of all the sensors toward 1.25–25 ppm 3H-2B was further tested (Figure 3b), which was transformed into a dot–line plot (Figure 3c). The 1Au/SnO_2_–WO_3_ nanoplate-based sensors exhibited an enhanced response for the whole detection range, indicating a good linear relationship. In terms of the sensing speed, the 1Au/SnO_2_–WO_3_ nanoplates showed a short response/recovery time of 25s and 11s, respectively (Figure 3d). Moreover, the 1Au/SnO_2_–WO_3_ nanoplate-based sensors exhibited a quick response and could repeatedly be tested when cycled between 3H-2B gas and ambient air (Figure S5), indicating good reproducibility. The 1Au/SnO_2_–WO_3_ nanoplate sensor had a higher baseline resistance, about 16 times higher than that of the WO_3_ sensor (Figure 3e). The high baseline resistance may have originated from the existence of a large amount of oxygen on the surface of the 1Au/SnO_2_–WO_3_ nanoplates.

Other than sensitivity and rapid response/recovery, selectivity is another important factor in practical applications. The sensors were tested using different interference gases (methanol, ethanol, toluene, n-hexane, benzaldehyde, isopropyl alcohol, acetone, ammonia, formaldehyde, and NO_2_) at 25 ppm at 140 °C. At a low working temperature (140 °C), no valid detection value could be obtained for acetone, ammonia, and formaldehyde. *Listeria monocytogenes* is a contagious food pathogen that exists in vegetables, fish, meat, and dairy products, causing fatal foodborne illness. 3-Hydroxy-2-butanone (32.2% abundance) and benzaldehyde (17.6% abundance) are the typical microbial volatile organic compounds produced by *Listeria monocytogenes*, which can be characterized as biomarkers [27]. The response of all sensors to 3H-2B was higher than that for other gases (Figure 3f). Among them, the response of the 1Au/SnO_2_–WO_3_ nanoplate sensors to 3H-2B was the highest and was 45.98 times that of benzaldehyde and 185 times that of methanol. The response to oxidizing gas NO_2_ could be ignored. The detection results of 3H-2B using different MOS-based sensors in recent years are summarized in Table 1. Accordingly, the 1Au/SnO_2_–WO_3_ nanoplate sensors used in the present study exhibited a highly sensitive, good selective, and rapid detection of 3H-2B at a low temperature, which is beneficial for the convenient detection of *Listeria monocytogenes*.

### 3.4. Sensing Mechanism

**Figure 4:**
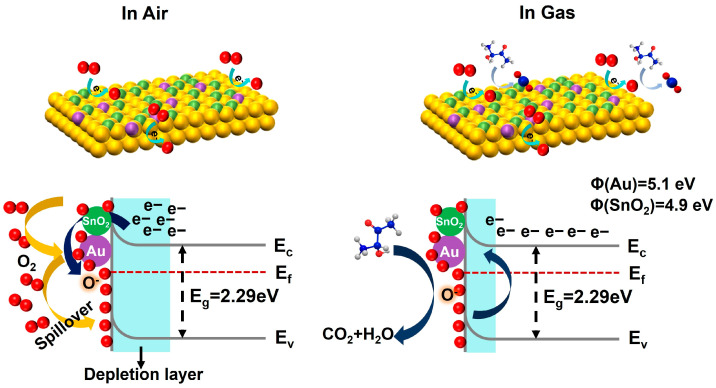
Schematic of the surface-sensing reaction of 1Au/SnO_2_–WO_3_ nanoplates toward 3H-2B and the corresponding band diagram of the sensing mechanism.

The 3H-2B-sensing mechanism of WO_3_-based sensors has been investigated in previous reports [26,27]. As illustrated in Figure 4, when n-type WO_3_ was exposed to air, an electron depletion layer was formed on the surface of WO_3_ due to the adsorption of oxygen molecules. Thereafter, when 3H-2B was introduced, the target gas reacted with the chemisorbed oxygen species, producing byproducts of acetic acid, 2,3-butanedione, and eventually H_2_O and CO_2_. Thus, this reaction process released the captured electrons and shortened the electron depletion layer, decreasing the resistance. The improved 3H-2B-sensing performance of the 1Au/SnO_2_–WO_3_ nanoplates could be ascribed to (i) the heterojunctions between the Au and SnO_2_ nanoparticles and the WO_3_ nanoplates, (ii) the spillover effect of catalytic Au nanoparticles, and (iii) sufficient oxygen vacancies. First of all, the differences in the work function of dominant WO_3_ (5.24 eV) and partially dispersed SnO_2_ (4.9 eV) formed an additional electron depletion layer, inducing an increase in the material resistance [45]. In addition, the introduction of Au nanoparticles remarkably improved the dissociation of oxygen molecules, resulting in the further expansion of the electron depletion layer. This was consistent with the observed differentiation in bulk resistance, where the base resistance of the 1Au/SnO_2_–WO_3_ nanoplates (180 MΩ) was larger than that of the WO_3_ nanoplates (11 MΩ) (Figure 3e). Furthermore, there were more active sites for the adsorption and oxidation of 3H-2B due to the presence of oxygen vacancies. The changes in the work function values, Fermi level, and band edge position for the 1Au/SnO_2_–WO_3_ nanoplates were determined using UV–vis absorption spectroscopy and ultraviolet photoelectron spectroscopy (Figure S6). The determined work functions stood at 4.77 and 4.39 eV respectively, pertaining to the WO_3_ and 1Au/SnO_2_–WO_3_ nanoplates. The disparity in the work functions suggested a higher electron population in the conduction band of the 1Au/SnO_2_–WO_3_ nanoplates compared with that of the WO_3_ nanoplates, thereby augmenting the adsorption capacity for oxygen species. Consequently, this facilitated an efficient electron exchange with gas molecules and promoted a more facile gas-sensing process.

### 3.5. Wireless Portable Sensor

**Figure 5:**
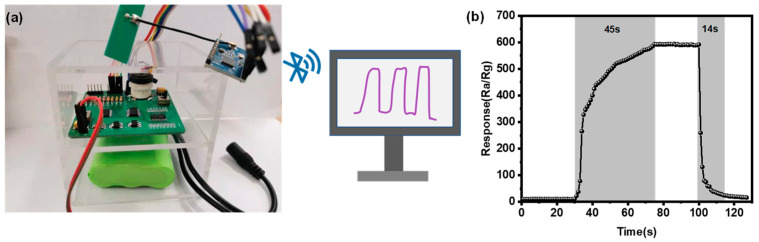
(**a**) Optical photograph of wireless portable sensor connected to a laptop via ZigBee. (**b**) Dynamic response and recovery curves displayed on the laptop when the portable sensor was exposed to 25 ppm 3H-2B.

In order to realize the real-time detection of 3H-2B, a wireless portable sensor based on the 1Au/SnO_2_–WO_3_ nanoplate gas sensor was developed and connected to a laptop via ZigBee, which provided the sensing information (Figure 5). By integrating the power module, electric heating wire temperature control module, series resistor selection module, wireless module, main control chip, and sensing module on an 8 cm × 8 cm PCB circuit board using integrated circuit technology, the volume of the gas detection device was significantly reduced, resulting in cost savings and reduced power consumption. The homemade portable sensor displayed a rapid sensing response toward 25 ppm 3H-2B at 140 °C, which was comparable with the sensing information obtained from the gas-response instrument. The feasibility of the on-site monitoring of *Listeria monocytogenes* by detecting the concentration of 3H-2B has been demonstrated [13,46]; thus, these results suggest that a portable gas sensor holds great potential for the rapid identification of foodborne pathogens.

## 4. Conclusions

In summary, a ternary heterostructure was prepared by decorating WO_3_ nanoplates with Au and SnO_2_ nanoparticles through a facial photochemical deposition method, and was employed as a sensing material for 3-hydroxy-2-butanone (3H-2B), a biomarker of *Listeria monocytogenes*. These 1Au/SnO_2_–WO_3_ nanoplate-based sensors exhibited an excellent response (R_a_/R_g_ = 662) to 25 ppm 3H-2B, which was 24 times higher than that of the pure WO_3_ nanoplates at 140 °C. Moreover, the 3H-2B sensor showed rapid response/recovery and high selectivity. These exceptional sensing performances could be attributed to the heterojunctions between the Au and SnO_2_ nanoparticles and the WO_3_ nanoplates, the spillover effect of the catalytic Au nanoparticles, and abundant oxygen vacancies. Furthermore, a wireless portable gas sensor equipped with the 1Au/SnO_2_–WO_3_ nanoplates was assembled and exhibited comparable detection values with the sensing information obtained from the gas-response instrument.

## Figures and Tables

**Table 1:** Comparison of the gas-sensing performance of MOS-based sensors to 3H-2B.

Sensing Materials	T (°C)	τres/τrecov (s)	3H-2B (ppm)	Response (R_a_/R_g_)	LOD (ppm)	Ref.
WO_3_	205	25/146	25	152	0.4	[39]
ZnO@Al_2_O_3_	300	27/34	50	37.2	10	[40]
Cr_2_O_3_/SnO_2_	240	9/4	50	280	0.02	[41]
Pt-doped SnO_2_	250	11/20	10	48.69	0.5	[42]
Pd–BiVO_4_	200	12/8	10	103.7	0.2	[43]
M–NiO NCs	120	49/52	50	302	0.5	[44]
WO_3_/Au	175	15/45	2.5	18.8	2.5	[26]
1Au/SnO_2_–WO_3_	140	25/11	25	662	1.25	This work

## Data Availability

Data are contained within the article and Supplementary Materials.

## Supplementary Materials

The following supporting information can be downloaded at: https://www.mdpi.com/article/10.3390/nano14080719/s1, Figure S1: TEM images and XRD patterns of WO_3_·H_2_O nanosheets; Figure S2: TEM images and XRD patterns of WO_3_·H_2_O nanoplates; Figure S3: TEM images and SEM images of Au–WO_3_ nanoplates, SnO_2_–WO_3_ nanoplates, and Xau/SnO_2_–WO_3_ nanoplates; Figure S4: XRD and XPS patterns of XAu/SnO_2_–WO_3_ nanoplates; Figure S5: Dynamic sensing response of the sensor based on 1Au/SnO_2_–WO_3_ nanoplates to 25 ppm 3H-2B at 140 °C; Figure S6: UV–vis absorption spectra, Tauc plots, and UPS spectra of WO_3_ and 1Au/SnO_2_–WO_3_ nanoplates; Table S1: The mass percentage of Au and Sn elements in XAu/SnO_2_–WO_3_ nanoplates; Table S2. The mass percentage of Au and Sn elements in XAu/SnO2-WO3 nanoplates. Note S1: Chemicals and Materials, Characterizations, and Gas-Sensing Measurements.
