# Supplementary material for: WO3 Nanoplates Decorated with Au and SnO2 Nanoparticles for Real-Time Detection of Foodborne Pathogens
Source: Nanomaterials (Basel). 2024 Apr 19;14(8):719. doi: 10.3390/nano14080719 (PMC11054436; doi:10.3390/nano14080719)
Supplement: Supplementary file 1 [file nanomaterials-14-00719-s001.zip › nanomaterials-2956436-supplementary.pdf]

nanomaterials

Supporting Materials

WO_3 Nanoplates decorated with Au and SnO_2 nanoparticles for Real Time Detection of Foodborne Pathogens

Xueyan Li [1], Zeyi Wu [1], Xiangyu Song [1], Denghua Li [2,*], Jiajia Liu [1,*] and Jiatao Zhang [1,3,4]

[1] School of Materials Science and Engineering, Beijing Key Laboratory of Construction-Tailorable Advanced Functional Materials and Green Applications, Beijing Institute of Technology, Beijing 100081, China; zhangjt@bit.edu.cn (J.Z.)
[2] Key Laboratory of Agricultural Information Service Technology of Ministry of Agriculture, Agricultural Information Institute of Chinese Academy of Agricultural Sciences, Beijing 100081, China
[3] School of Chemistry and Chemical Engineering, MIIT Key Laboratory of Medical Molecule Science and Pharmaceutical Engineering, Beijing Institute of Technology, Beijing 100081, China
[4] MOE Key Laboratory of Cluster Science, Beijing Institute of Technology, Beijing 100081, China
* Correspondence: lidenghua@caas.cn (D.L.); liujiajia@bit.edu.cn (J.L.)

Chemicals and Materials:

Sodium tungstate ($Na_2WO_4 \cdot 2H_2O$, 98%) was purchased from Shanghai Adamas Reagent Co., LTD. L(+)-Lactic acid ($C_3H_6O_3$, 99%), Chloroauric acid ($HAuCl_4$, 99%), 3-hydroxy-2-butanone ($C_4H_8O_2$, 98%) were purchased form Shanghai Aladdin Biochemical Technology Co., LTD. Hydrochloric acid (HCl, 36% ~ 37%, w/w) was purchased from Sinopharm Group Chemical reagent Co., LTD. Stannous chloride ($SnCl_2$, 99%) was purchased from Beijing Tongguang Fine Chemical Company. All reagents were used without any further purification.

Characterizations.

The morphology of the samples was analyzed using field emission scanning electron microscopy (FESEM, Regulus8230). Low-resolution transmission electron microscopy (TEM) images were acquired on a HITACHI H-7650 (accelerating voltage of 80 kV) electron microscopy. High-resolution transmission electron microscopy (HRTEM) characterization and energy dispersive X-ray spectroscopy (EDS) analysis were operated on a transmission electron microscopy (FEI Tecnai G2 F20 S-Twin, an acceleration voltage of 200 kV) equipped with X-ray energy-dispersive spectroscopy detector. The XRD patterns were obtained by using a Bruker D8 multiply crystals X-ray diffractometer with Cu Kα radiation (5 °·min-1). The XPS spectra were recorded on a PerkinElmer Physics PHI 5300 spectrometer using mono-chromatic Al Kα radiation (1486.7 eV). Optical absorption spectra were recorded using Shimadzu UV3600 spectrophotometer. Ultraviolet photoelectron spectrometer (UPS) was taken on a Thermo ESCALAB 250Xi spectrometer, where the HeI energy of the excitation source is 21.22 eV, and the bias voltage of -10 eV is added during the test. Electron Paramagnetic Resonance (EPR) spectra were obtained on a Burker A300-10/12 electron paramagnetic resonance spectrometer.

Gas-Sensing Measurement.

The obtained sample was mixed with a certain amount of deionized water to form a paste. Then, the paste was applied to the surface of a ceramic tube, which equipped with Pt-Au electrodes and a Ni-Cr alloy wire as a heater. Finally, the six wires of the ceramic tube were welded onto base and aged at 260 °C for three days. A gas response instrument (WS-30B, Weisheng Ltd., Zhengzhou, China) was used to measure the gas-sensing characteristics with static gas distribution method. For volatile organic compounds detection, a certain volume of liquid was injected into the test chamber on a heating platform. The NO_2 concentration in the 18 L sensing chamber was controlled by injecting certain

volumes of 1800 ppm NO_2 diffusing in N_2 with a syringe. When the gas sensor resistance was stable, the lid was removed, and the mixed gas diffused away. The resistance ratio between the gas sensor in air (R_a) and target gas (R_g) was calculated as the gas response, where R_g/R_a and R_a/R_g were for oxidizing gas and reducing gases, respectively. The response/recovery time is defined as the time for the variation in gas response to reach 90% of the equilibrium value after a test gas was injected or released.

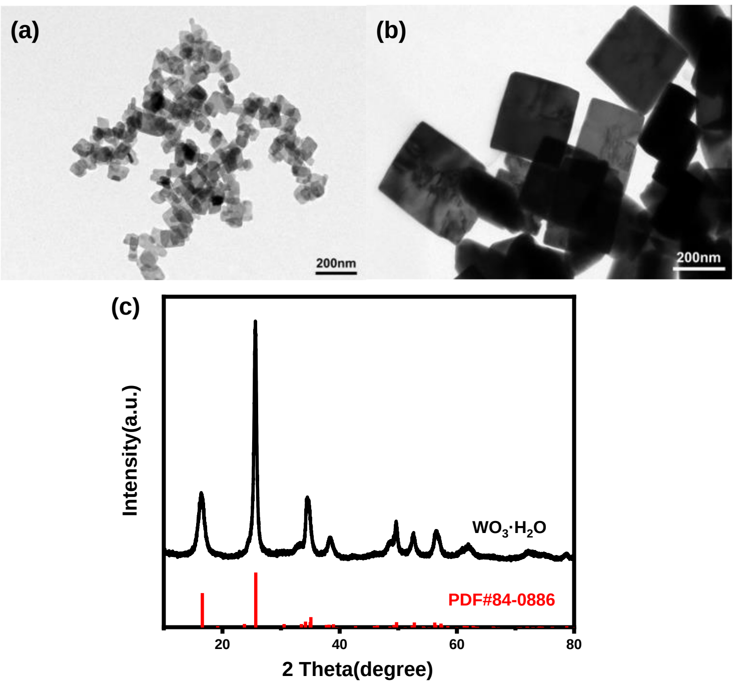

Figure S1. (a, b) TEM images of pre-hydrothermal products and $WO_3·H_2O$ nanosheets; (c) The XRD patterns of $WO_3·H_2O$ nanosheets.

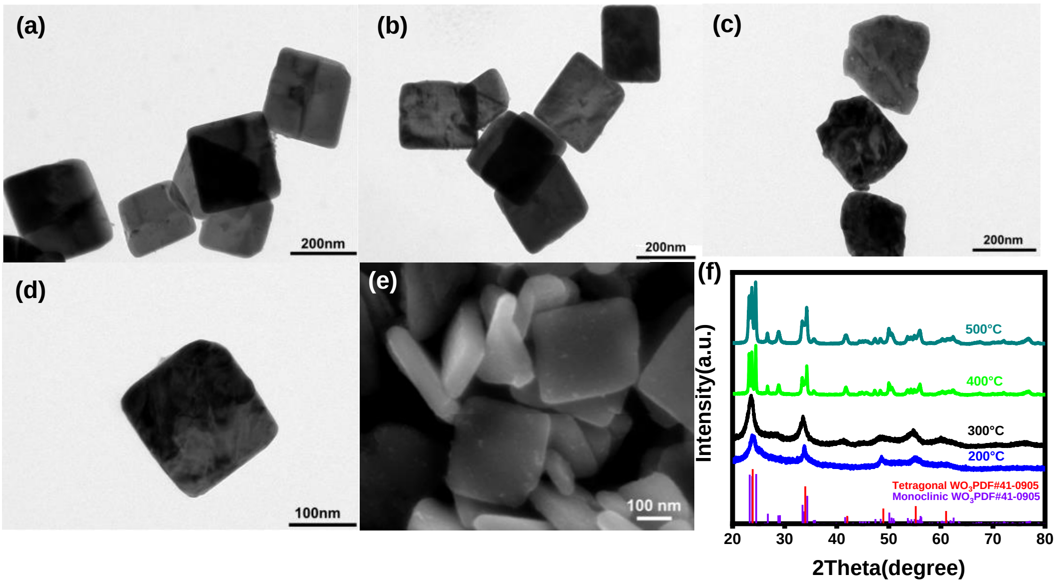

Figure S2. (a) TEM image of WO_3 nanoplates after 200°C calcination ; (b) TEM image of WO_3 nanoplates after 300°C calcination ; (c) TEM image of WO_3 nanoplates after 500°C calcination ; (d-e) TEM and SEM images of WO_3 nanoplates after 400°C calcination; (f) The XRD patterns of WO_3 nanoplates after 200°C, 300°C, 400°C and 500°C calcination.

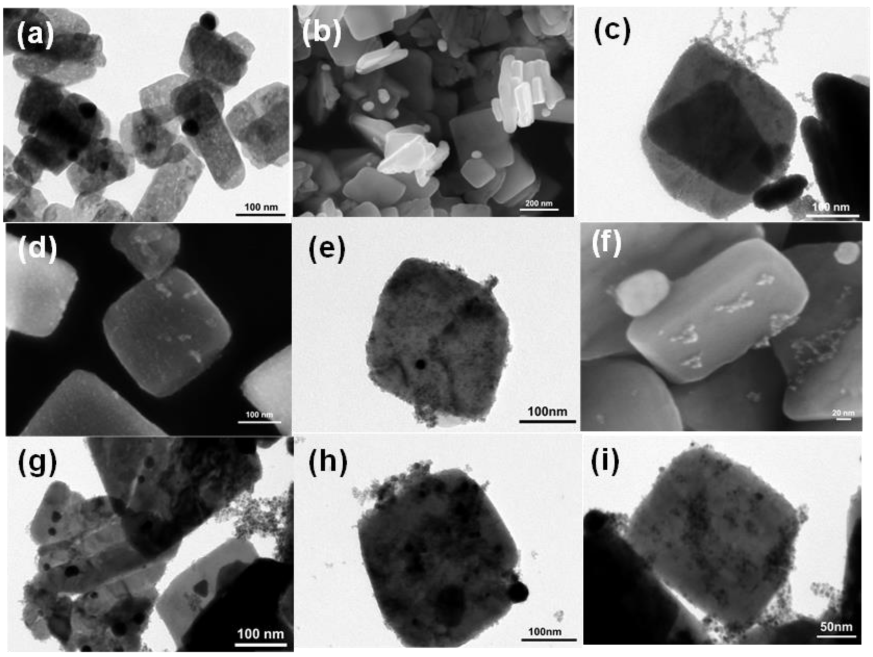

Figure S3. (a-b) The TEM and SEM images of Au-WO_3 nanoplates; (c-d) The TEM and SEM images of SnO_2-WO_3 nanoplates; (e-f) The TEM and SEM images of 1Au/SnO_2-WO_3 nanoplates; (g) The TEM images of 0.5Au/SnO_2-WO_3 nanoplates; (h) The TEM images of 0.75Au/SnO_2-WO_3 nanoplates; (i) The TEM images of 1.25Au/SnO_2-WO_3 nanoplates.

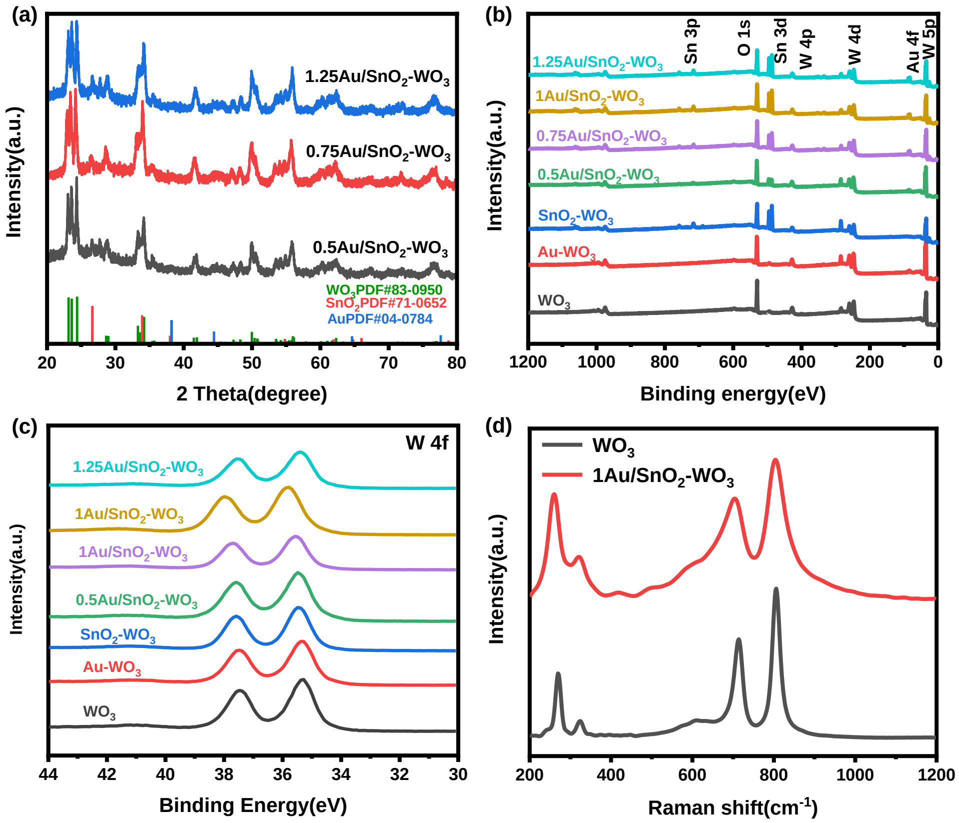

Figure S4. (a) The XRD patterns of XAu/SnO_2-WO_3 nanoplates; (b) The survey XPS patterns of WO_3 nanoplate-based and XAu/SnO_2-WO_3 nanoplate-based sensors; (c) XPS spectra of W 4f; (d) Raman spectra of WO_3 nanoplates and 1Au/SnO_2-WO_3 nanoplates.

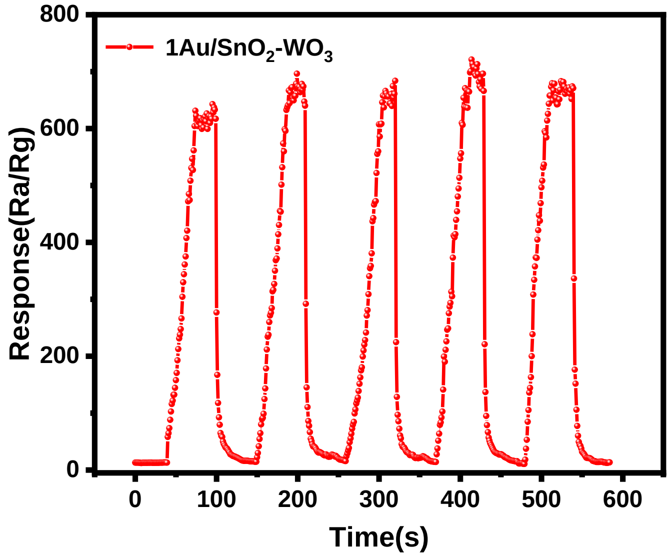

Figure S5. Dynamic sensing response of the sensor based on 1Au/SnO_2-WO_3 nanoplates to 25 ppm 3H-2B at 140°C.

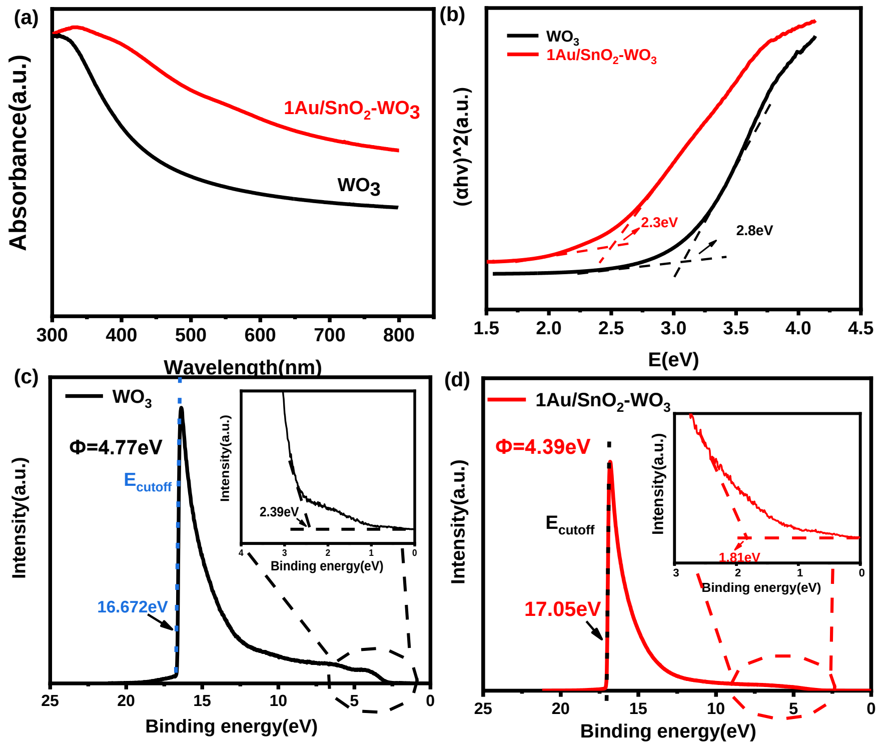

Figure S6. (a) UV−vis absorption spectra, (b) Tauc Plot of WO_3 nanoplates and 1Au/SnO_2-WO_3 nanoplates; UPS spectra of (c) WO_3 nanoplates and (d) 1Au/SnO_2-WO_3 nanoplates.

Table S1. Samples prepared in this work.

**Sample**	Preparation method	Materials in deposition	
		$HAuCl_4$ (5 mg/mL)	$SnCl_2$ (5 mg/mL)
**WO_3**	Hydrothermal and calcination	-	-
Au-WO_3	Photochemical deposition	1 mL	-
SnO_2-WO_3	Photochemical deposition	-	1 mL (+2g KIO_3)
0.5Au/SnO_2-WO_3	Photochemical deposition	0.5 mL	0.5 mL
0.75Au/SnO_2-WO_3	Photochemical deposition	0.75 mL	0.75 mL
1Au/SnO_2-WO_3	Photochemical deposition	1 mL	1 mL
1.25Au/SnO_2-WO_3	Photochemical deposition	1.25 mL	1.25 mL

Table S2. The mass percentage of Au and Sn elements in XAu/SnO_2-WO_3 nanoplates.

**Sample**	**Au(wt%)**	**Sn(wt%)**
Au-WO_3	1.95	-
SnO_2-WO_3	-	19.56
0.5Au/SnO_2-WO_3	5.21	10.34
0.75Au/SnO_2-WO_3	5.43	14.57
1Au/SnO_2-WO_3	5.71	19.42
1.25Au/SnO_2-WO_3	6.61	19.55
